# Phylogenomic diversity of clinical *Saccharomyces cerevisiae* and the prevalence of probiotic-derived isolates in a tertiary care center in Hungary

**DOI:** 10.1128/spectrum.02750-25

**Published:** 2025-11-26

**Authors:** Andrea Harmath, Bálint Németh, István Pócsi, László Majoros, Walter P. Pfliegler, Renátó Kovács

**Affiliations:** 1 Department of Medical Microbiology, Faculty of Medicine, University of Debrecen 37599, Debrecen, Hungary; 2 Doctoral School of Pharmaceutical Sciences, University of Debrecen 37599, Debrecen, Hungary; 3 Doctoral School of Nutrition and Food Sciences, University of Debrecen 37599, Debrecen, Hungary; 4 Department of Molecular Biotechnology and Microbiology, Faculty of Science and Technology, University of Debrecen 37599, Debrecen, Hungary; 5 HUN-REN-UD Fungal Stress Biology Research Group, HUN-REN Hungarian Research Network, Debrecen, Hungary; University of Wisconsin-Madison Laboratory of Genetics, Madison, Wisconsin, USA

**Keywords:** probiotic, *Saccharomyces boulardii*, susceptibility, phylogenomics, yeast, invasivity

## Abstract

*Saccharomyces cerevisiae* is traditionally considered a non-pathogenic microbe; however, recent evidence has established its role as an opportunistic fungal pathogen. The probiotic subtype, var. “*boulardii*,” is widely administered for the prevention and treatment of gastrointestinal disorders. Here, we evaluated 46 clinical *S. cerevisiae* isolates obtained from a tertiary care center in Hungary. Conventional species identification was complemented by multiplex PCR fingerprinting and phylogenomic assignment within a global genomic framework. In addition to these approaches, antifungal susceptibility testing, agar invasiveness, and killer activity assays were performed to provide a comprehensive characterization. Of the isolates, 22 (48%) clustered within the probiotic subclade, predominantly originating from respiratory tract samples, while 15 (33%) corresponded to clades associated with commercial baker’s yeasts. Probiotic-derived isolates were significantly associated with pediatric patients (<18 years; OR = 15.92, *P* = 0.004) and with prior probiotic administration (*S*. “*boulardii”*: OR = 11.0, *P* = 0.003). Caspofungin and anidulafungin retained consistent *in vitro* activity, whereas amphotericin B and micafungin exhibited broader MIC distributions. Sporadic isolates from wine and ale yeast clades highlighted the heterogeneous origins of clinical *S. cerevisiae*. Regardless of their phylogenomic relationships, isolates generally lacked invasiveness or complex colony morphologies that are often regarded as potential virulence traits. This study demonstrates that clinical *S. cerevisiae* isolates frequently derive from probiotic or industrial lineages, with distinct patient associations. Integrating phylogenomics, antifungal profiling, and clinical parameters, all obtained in a single location, provides critical insights into the epidemiology, pathogenic potential, and therapeutic management of this potentially emerging fungal pathogen.

**IMPORTANCE:**

*Saccharomyces cerevisiae* is best known for its beneficial role in beer, bread, and wine production and is generally considered a harmless microbe. A specific subtype, *S. cerevisiae* var. “*boulardii,”* is even marketed worldwide as a probiotic for the prevention and treatment of various gastrointestinal disorders. However, in hospitalized patients—particularly in children and immunocompromised individuals—this yeast can act as an opportunistic pathogen. In our study, nearly half of all clinical isolates belonged to the probiotic lineage, and their occurrence showed a strong correlation with prior probiotic use. By integrating genome sequencing, antifungal susceptibility testing, and patient data, we demonstrate that yeasts considered safe in food and probiotic products may nevertheless cause infections in vulnerable individuals. These findings highlight the need for clade-level molecular genetic typing of *Saccharomyces* samples, cautious probiotic administration, and sustained clinical vigilance.

## INTRODUCTION

The yeast *Saccharomyces cerevisiae* has long been considered a non-pathogenic microorganism, playing a crucial role in the food industry and serving widely as a model organism in molecular biology. However, accumulating clinical evidence in recent years suggests that this species—particularly the probiotic subtype *S. cerevisiae* var. “*boulardii”*—can act as an opportunistic pathogen, especially in immunocompromised or critically ill patients (1, 2). *S. cerevisiae* var. “*boulardii”* is a widely available probiotic, commonly used for the prevention and management of antibiotic-associated diarrhea and *Clostridioides difficile* infection (3, 4), and it is often erroneously reported as a separate species in literature (as *S. boulardii*). Although its use is generally considered safe in healthy populations, cases of invasive infections linked to this probiotic have been reported, primarily among patients receiving broad-spectrum antibiotics, those in intensive care units, and individuals with central venous catheters (e.g., references 4–10).

Current epidemiological data reveal a rising incidence of bloodstream infections caused by emerging fungal pathogens, including *S. cerevisiae*, often exhibiting heterogeneous antifungal resistance profiles (8, 11, 12). This trend highlights the importance of precise identification (13) and comprehensive antifungal susceptibility testing. While echinocandins and amphotericin B retain activity against *S. cerevisiae*, the lack of species-specific clinical breakpoints further complicates therapeutic management (14, 15).

*S. cerevisiae* is a heterogeneous, globally distributed species comprising numerous described clades, many of which are adapted to specific environmental niches or fermentation-related conditions, leading to a great variability in tolerating various stress factors (16–20). Several clades have been shown to contain human isolates based on phylogenomic data, and the literature to date has generally used the terms “clinical isolate” and “human isolate” interchangeably (17, 19, 21). However, human isolates may not necessarily show pathogenic adaptations, and their relationship to host health is often unclear (8, 22). Often, the lack of detailed patient data complicates efforts to determine whether the isolates in question are merely transient or long-term colonizers of the human body, or true pathogens causing disease—especially since medium- or long-term follow-up studies of *S. cerevisiae* isolates from human hosts have not yet been published. The role that each clade may play in this regard remains an open question, especially as recent studies have revealed the characteristics and diversity of clades to an unprecedented extent (16, 17). Human isolates sequenced to date predominantly fall into clades associated with commonly used fermentation strains (e.g., the Mixed Origins 1-2 clades, which include baker’s yeasts), clades primarily composed of clinical isolates (e.g., the US Clinical 1–3 clades), the probiotic yeast subclade (*S. cerevisiae* var. “*boulardii*,” which clusters within the West European wine clade), or the distinct and local French Guiana human clade (16, 17, 23).

A deeper understanding of the pathogenicity, niche preferences, and resistance mechanisms of *S. cerevisiae*—both at the species level and across its diverse clades—is critical for assessing its potential as an emerging pathogen, ensuring the safe use of yeast probiotics, and developing effective antifungal treatment strategies, particularly for vulnerable patient populations. In this study, we analyzed and compared human isolates of the species collected over an 8-year period at a single university hospital in Debrecen, Hungary, supported by detailed patient data. We employed a large-scale phylogenomic approach for clade- and strain-level identification, complemented by antifungal susceptibility testing, killer activity assays, and agar invasiveness assays. This analysis allowed us to investigate how *S. cerevisiae* colonizes or infects different anatomical sites and patient groups, examine how phenotypic traits and clinical data correlate with clade origin, and explore whether clinical isolates can be directly linked to commonly used yeast strain groups found in various products.

## MATERIALS AND METHODS

### Patient and isolate information

Our study was conducted at a Hungarian Clinical Centre affiliated with the University of Debrecen, which has more than 1,700 inpatient beds. This study summarizes infections caused by *S. cerevisiae,* including its probiotic subtype, *S. cerevisiae* var. “*boulardii*.” A total of 46 *Saccharomyces* isolates were analyzed, collected randomly between 2015 and 2023: 13 from throat swabs, 11 from cervical specimens or vagina, 14 from bronchial samples, trachea, sputum, or nose, 4 from wound swabs, 3 from bloodstream samples, and 1 from a cornea sample (Table S1). All isolates were routinely identified using matrix-assisted laser desorption/ionization time-of-flight mass spectrometry (MALDI-TOF MS). Culturing was performed on Sabouraud dextrose agar plates incubated at 37°C. All isolates are stored as duplicates in the culture collections of the Department of Medical Microbiology and Department of Molecular Biotechnology and Microbiology as frozen stocks in yeast extract peptone dextrose (YPD) (2% glucose, 2% peptone, and 1% yeast extract) supplemented with 30% (vol/vol) glycerol. Clinical data were retrieved from patient medical records and included demographic information, clinical presentation (e.g., isolation conditions, healthcare-associated risk factors, underlying comorbidities, and treatment), and 30-day mortality. Selected laboratory results were also recorded, including white blood cell count, neutrophil granulocyte count, lymphocyte count, C-reactive protein, and procalcitonin levels.

### Screening probiotic origin using multiplex PCR

Isolates identified as *S. cerevisiae* by MALDI-TOF were further subtyped using multiplex PCR (13) as a means of rapid screening of the probiotic subtype of the species by their characteristic banding pattern in the fingerprinting assay while also enabling a phylogenomics-backed validation of the fingerprinting method. Genomic DNA extraction was performed according to the protocol described by Hanna and Xiao (24), and DNA concentrations were standardized to 100 ng/µL for all isolates. Genomic DNA samples were stored in 1× Tris-EDTA buffer at −20°C. Subtyping was based on the use of the primer sets designed to amplify interdelta elements, microsatellites of the genes *YLR177w*, *YOR267c*, and the internal transcribed spacer region as described by Imre et al. (13).

### Antifungal susceptibility testing

*Saccharomyces* isolates were pre-cultured in liquid Sabouraud dextrose broth and subcultured on solid Sabouraud dextrose agar at 37°C for antifungal susceptibility testing. Susceptibility to fluconazole, amphotericin B, anidulafungin, caspofungin, and micafungin (all from Merck, Budapest, Hungary) was assessed in YPD (2% glucose, 2% peptone, and 1% yeast extract) using the broth microdilution method, following the Clinical and Laboratory Standards Institute M27-A3 guideline (25). According to the M27-A3 guideline, the microdilution assay should be performed in RPMI-1640 medium (with L­-glutamine and without bicarbonate, pH 7.0 with [3-(N-morpholino) propanesulfonic acid] buffer; Merck, Budapest, Hungary). However, most of our isolates did not exhibit growth in this medium. Therefore, minimum inhibitory concentrations (MICs) were determined using YPD medium instead ([8], similarly to earlier observations in reference 22). The tested antifungal concentration ranges were 0.06–32 mg/L for fluconazole, 0.03–16 mg/L for amphotericin B, and 0.004–2 mg/L for echinocandins. For all the antifungals, only MIC_50_ and MIC_90_ values were reported (26). In the case of MIC determination, partial inhibition (≥50% growth reduction vs growth control) was used for fluconazole and echinocandins, while complete inhibition (100% growth reduction) was applied for amphotericin B.

### Colony morphology and invasiveness

Colony phenotypes were examined by spotting 10 µL of overnight cultures—grown on YPD at 30°C and adjusted to an OD_₆₀₀_ of 0.1—onto solid media. The medium used was YPD (2% glucose, 2% peptone, 1% yeast extract, and 3% agar) (27). Cultures were plated onto media in vented plastic Petri dishes and incubated for 10 days at 37°C, with the agar surface facing downward. After incubation, colony morphology was evaluated visually. To assess invasiveness, colonies were gently rinsed under tap water and examined using transillumination (uniform white light) to determine the extent of agar penetration. Photos were taken of the dishes with a digital camera, and the software ImageJ was used to determine the intensity value distribution of pixels in three different empty areas of the medium. A weighted average was calculated as a “darkness” value using the number of pixels and their intensities. This was used as a background darkness value after correcting for the total number of pixels. The same measurements and calculations were performed for the washed-off colonies in triplicate. Invasive areas’ darkness values were divided by the averaged background darkness values (similarly to 27). With this method, a darkness value of, for example, 1.5 means that the washed-off colony is 1.5 times darker than the empty medium due to the presence of invasive cells in the medium that block some of the light during transillumination.

### Killer activity assay

We tested the so-called killer toxin production by the clinical isolates using a sensitive control strain (NCYC 1006). Yeast killer toxins specifically act on other yeasts and are, apart from rare examples, only produced when a strain is infected with the killer virus. Killer virus infection and toxin production are prevalent, especially among members of the wine yeast clades (28). Killer activity tests were performed using the halo assay on YPD agar plates supplemented with 0.003 g/L methylene blue, buffered to pH 4.5 with citrate-phosphate buffer. The sensitive strain was set to 1 MacFarland density, and 100 µL suspensions were inoculated around the tested colonies (~200,000 CFU). Plates were incubated for 4 days at 25°C and 37°C, and killer activity was registered when a halo of growth inhibition in the case of the sensitive control was visible (28).

### Phylogenomics

A reference-based mapping and SNP-based phylogenomic approach was used to cluster the clinical isolates into the described clades of the species. To enable comparisons with previously published clades, mosaic groups, and even strains to an unprecedented level, we relied on the Compendium of *Saccharomyces* genomes (B. Németh, A. Imre, A. Harmath, H. V. Rácz, P. Oláh, R. Kovács, I. Pócsi, and W. P. Pfliegler, submitted for publication). The isolates from the Debrecen clinics were included in a large-scale phylogeny of nearly 4,500 *S*. *cerevisiae* genomes. Their cladal origins were determined based on clustering with known members of clades described by Loegler et al. (16). As the phylogeny included all previously published genomes available for commercial strains, we were also able to determine whether a given clinical isolate showed strain-level identity to any previously described yeast.

The DNA isolated as described above was subjected to short-read sequencing either by Novogene (Germany) as a paid service or by the Core Facility of the University of Debrecen. In the latter case, library preparation was performed using tagmentation with the Nextera DNA Flex Library Prep Kit (Illumina, San Diego, CA, USA) according to the manufacturer’s protocol. Sequencing was performed using 150 bp paired-end reads on an Illumina NextSeq 500 system, with approximately 50–80× coverage of the nuclear genome. In the case of Novogene, sequencing was performed using paired-end 150 bp technology on an Illumina NovaSeq X Plus system. During library preparation, genomic DNA was fragmented to an average size of 350 bp, and size selection was carried out using sample purification beads. Raw reads of new samples were deposited to NCBI SRA under BioProject PRJNA1313639. The Illumina FASTQ sequencing files were trimmed and filtered using fastp for further analysis (29). We mapped Illumina reads to a concatenated reference panel of eight *Saccharomyces* species (*S. arboricola*, *S. cerevisiae*, *S. eubayanus*, *S. jurei*, *S. kudriavzevii*, *S. mikatae*, *S. paradoxus*, and *S. uvarum*) downloaded from GenBank to avoid introgressed regions in several clades and non-*cerevisiae* subgenomes in hybrids to skew phylogenomic positions. Mapping was performed using the mem option of BWA 0.7.17 (30). Sorted BAM files were obtained using Samtools 1.7 (31). Picard-tools 2.23.8 was used to mark duplicated reads (32, 33). Using BAM files, local realignment around indels and joint variant calling and filtering for the strains and isolates were performed with GATK 4.1.9.0 (32, 33), with regions annotated in the S288c reference as centromeric regions, telomeric regions, or LTRs excluded. First, genomic VCF files were obtained with the Haplotype Caller for the *S. cerevisiae* chromosomes of the concatenated reference, and joint genotyping of the gVCF files was applied. Following the joint calling, only SNPs or only indels (insertions and deletions) were selected in the resulting VCF files. SNPs were filtered according to the parameters as follows: QD < 5.0; QUAL < 30.0; SOR > 3.0; FS > 60.0; MQ < 40.0; MQRankSum < −12.5; and ReadPosRankSum < −8.0. INDELS were filtered according to the parameters QD < 5.0; QUAL < 30.0; FS > 60.0; and ReadPosRankSum < −20.0 (34). Indels were then left-aligned. For the final VCF files, indels and SNPs were merged, filtered, and non-variant sites were removed. The multisample .vcf was converted to .gds file with the snpgdsVCF2GDS function of SNPrelate (35, 36). Then, a dissimilarity matrix was created using the snpgdsDiss function of SNPrelate. The matrix was imported into the ape 5.8-1 package (37), creating a neighbor-joining tree, and iTOL was used to visualize the output (38). Clades and clade colors were taken from Loegler et al. (16). Heterozygous positions of the genomes were exported using BCFTools’ (39) -stats option; the number of heterozygous INDELs and SNPs was compared to the length of the full *S. cerevisiae* genome and given as a percentage.

### Statistical analysis

Univariable analysis was performed to reveal factors associated with cladal origin. Categorical variables were analyzed using Fisher’s exact test. In the case of continuous variables, a logistic regression model was used. Data analysis was performed using GraphPad Prism software (version no.: 10.1.1). Results were considered significant if the *P*-value was <0.05.

## RESULTS

### Isolate collection at the Clinical Centre of the University of Debrecen

A total of 46 *Saccharomyces* isolates collected at the Clinical Centre during this study were analyzed. Demographic and clinical characteristics of the patients, along with the results of major laboratory investigations, were recorded. Two isolates originated from the same patient, while the remaining ones were from individual hosts (Table S1). Among the isolates, seven had previously been characterized in detail by our research group in terms of phenotype, virulence in *Galleria* model, and interactions with immune and epithelial cells (DE27020, DE35762, DE3912, DE42533, DE42807, DE45866, and DE6507) (8, 22). Of these, two were used in experimental mouse infection, and the deletion of the *HMX1* gene was studied to assess its effect on virulence (DE35762; DE6507) (40).

The isolates were preserved in our culture collection and subjected to DNA isolation. Multiplex PCR fingerprinting was performed on all strains to assess whether any could be traced back to the probiotic *Saccharomyces cerevisiae* var. “*boulardii”* yeast using this rapid method. This also provided an opportunity to evaluate the reliability of this rapid typing technique in light of the phylogenomic analysis described below, as the method had originally been developed using a limited number of clinical isolates and no whole-genome data (13). Isolates of probiotic origin exhibit a distinct banding pattern in this analysis, characterized by three specific regions comprising six bands grouped as follows: 600–850 bp, 210–270 bp, and 100–140 bp, along with an additional band of less than 75 bp. This characteristic pattern was observed in 22 cases, indicating the probiotic origin of nearly half of the isolates analyzed (Fig. S1).

**Fig 1:**
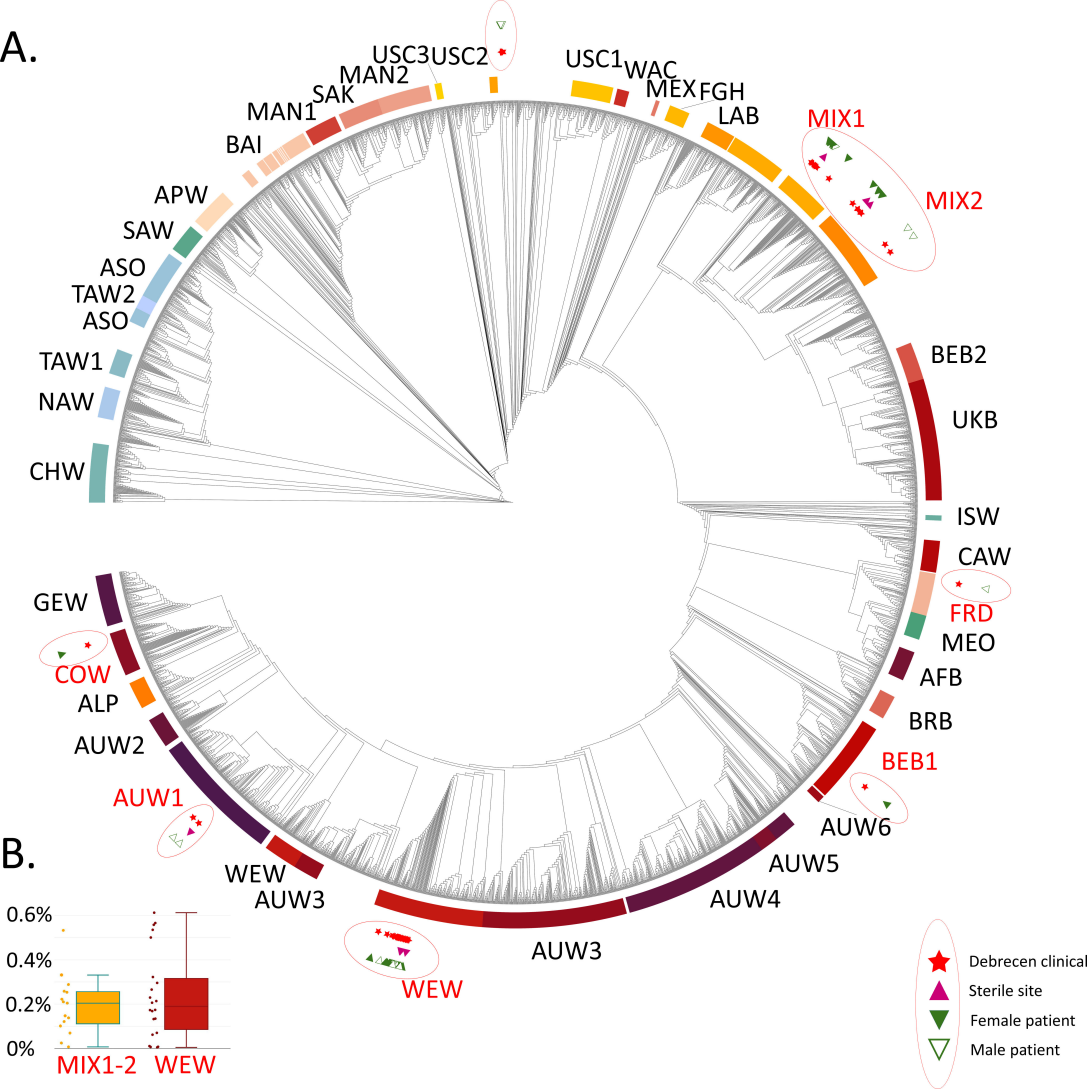
(**A**) Phylogenomic dendrogram of 4,475 *S*. *cerevisiae* genomes highlighting the origin of clinical isolates analyzed in this study. The analyzed clinical isolates’ positions on the dendrogram are marked with red stars inside red ellipses encircling each clade or group. Purple triangles mark isolates recovered from normally sterile anatomical sites. Full and empty green triangles mark female and male patients, respectively. Clades to which isolates belong are highlighted in red; others are in black font. A PDF version with searchable text of the tree with all genome names is included in Fig. S2. Clade abbreviations are as follows (16): MIX1, Mixed Origins 1; MIX2, Mixed Origins 2; FRD, French Dairy; BEB1, Belgian Beer 1; WEW, West Europe Wine; AUW1, AU Wine 1; and COW, Commercial Wine. (**B**) Box plots showing the percentage of heterozygous variant sites of the total *S. cerevisiae* genome for the Mixed Origins clades (MIX1–2) clinical genomes of this study and for *the S. “boulardii”* clinical genomes of this study (WEW). A horizontal line shows the median value, whiskers extend to the minimum and maximum values, and individual data points are shown.

Next, a reference-based whole-genome sequencing analysis incorporating allele calling and phylogenomics was applied to our isolate collection. Our study made use of the recently finished Compendium of *Saccharomyces* genomes, meaning that the 46 isolates were included in a phylogeny encompassing 4,475 unique genomes of *S. cerevisiae* and hybrids with an *S. cerevisiae* parent. This enabled comparison of the samples with all previously published and sequenced clades, including clinical isolates from earlier studies, and allowed for a more detailed assessment of the diversity within our collection than is achievable using fingerprinting PCR alone. Importantly, all isolates identified as *S. cerevisiae* var. “*boulardii”* with multiplex fingerprinting clustered unequivocally with members of the probiotic subclade (Fig. 1A; Fig. S1; Table S1). None of the isolates within this subclade exhibited a divergent banding pattern, thereby corroborating both the specificity and sensitivity of the fingerprinting assay.

In broth microdilution assays performed on all 46 isolates, amphotericin B MICs ranged from 2 to 16 mg/L, with MIC_50_ and MIC_90_ values of 8 and 16 mg/L, respectively. Fluconazole MICs ranged from 4 to 32 mg/L, with both MIC_50_ and MIC_90_ at 32 mg/L. For anidulafungin, MICs ranged from 0.0625 to 0.5 mg/L, with MIC_50_ and MIC_₉₀_ values of 0.25 and 0.5 mg/L, respectively. Micafungin MICs ranged from 0.0625 to 0.5 mg/L (MIC_50_/MIC_₉₀_ = 0.125/0.5 mg/L), while caspofungin MICs ranged from 0.015 to 0.125 mg/L (MIC_50_/MIC_₉₀_ = 0.0625/0.125 mg/L) (Table 1). Given the relatively high number of isolates belonging to the probiotic and baker’s yeast clades, MIC_50_ and MIC_90_ values were also determined separately for these groups, as detailed below.

**TABLE 1:** MIC_50_ and MIC_90_ values when all samples in this study were taken into account, and the same values for only the probiotic-derived and only the baker’s yeast-derived isolates in the case of the five antimycotics used in this study

	Amphotericin B	Fluconazole	Anidulafungin	Micafungin	Caspofungin
All isolates (*n* = 46)					
MIC_50_ (mg/L)	8	32	0.25	0.125	0.0625
MIC_90_ (mg/L)	16	32	0.5	0.5	0.125
Probiotic isolates (*n* = 22)					
MIC_50_ (mg/L)	4	32	0.25	0.125	0.0625
MIC_90_ (mg/L)	8	32	0.25	0.125	0.0625
Baker’s yeast isolates (*n* = 15)					
MIC_50_ (mg/L)	16	32	0.25	0.5	0.0625
MIC_90_ (mg/L)	16	32	0.5	0.5	0.125

Killer activity and invasivity were sporadically observed among the 46 samples (in the case of one and three isolates, respectively) and at 37°C on YPD plates. All samples showed similar entire, smooth colony morphologies, including the invasive isolates (Table S1).

### Clinical isolates related to the probiotic yeast

The phylogenomic analysis identified various clades or subclades containing clinical isolates from the University of Debrecen Clinical Centre, as well as samples with unclear phylogenomic relationships. Of the 46 *S. cerevisiae* isolates included in this study, 22 (48%) were classified as probiotic in origin (clustering into the probiotic yeast subclade of the West Europe Wine clade), while 24 (52%) were non-probiotic (Fig. 1; Fig. S1 and S2; Table S1). While all *S. “boulardii”* isolates formed a single cluster in the phylogenomic analysis (together with other *S. “boulardii”* from the literature), their heterozygosity differed notably, showing a bimodal distribution of low-to-medium heterozygosity isolates (0.0049%–0.3223%) and more highly heterozygous ones (0.5006%–0.6126%) (Fig. 1B; Table S1).

Patients with probiotic-origin isolates were significantly more likely to be under 18 years of age (41% vs 4%, OR 15.92, 95% CI 2.42–180.8, *P* = 0.004). Monomicrobial infections were observed in 52% of cases, similarly distributed between the groups. Healthcare-associated risk factors, including intensive care unit admission (33%), invasive mechanical ventilation (35%), central venous catheter use (54%), and prolonged hospital stay (>7 days; 61%), were common but did not show statistically significant associations with probiotic origin. The administration of probiotics in general, and *S. cerevisiae* var. “*boulardii”* specifically, was significantly associated with probiotic-origin isolates. Probiotic use was reported in 59% of cases in the probiotic group vs 13% in the non-probiotic group (OR 10.11, 95% CI 2.46–37.48, *P* = 0.0021). Similarly, *S. cerevisiae* var. “*boulardii”* administration was significantly more frequent among patients with probiotic-origin isolates (50% vs 8%, OR 11, 95% CI 2.15–53.5, *P* = 0.0031). Laboratory parameters revealed that white blood cell count was significantly higher in patients with probiotic-origin isolates (mean: 13.3 vs 8.2 giga/L; OR 1.24, 95% CI 1.03–1.49, *P* = 0.0211). Thirty-day mortality occurred in 20% of patients, with no significant difference between groups (*P* = 0.718). Patient data and analysis are summarized in Table 2. Regarding antimycotic tolerance tests, none of the probiotic isolates were able to grow in RPMI medium; therefore, the tests were carried out in YPD medium. Overall, MIC_50_ and MIC_90_ values for the 22 probiotic isolates were similar to those determined for the complete collection. Probiotics showed lower MIC_50_ and MIC_90_ values for Amphotericin B (4 and 8 mg/L, respectively) and a lower MIC_90_ for echinocandins as detailed in Table 1; however, these differences were mostly only one concentration step below those determined for all 46 isolates. A single isolate, DE33294, from a wound, among the 22 probiotic-related *S. cerevisiae,* showed notable invasivity in YPD agar medium.

**TABLE 2:** Patient statistics comparing isolates of the probiotic subclade with other phylogenomic groups

Variables	Total	Probiotic origin	Non-probiotic origin	Odds ratio	95% confidence intervals	*P*-value
	46 (100%)	22 (48%)	24 (52%)			
Demographic						
Age						
<18 years	10 (22%)	9 (41%)	1 (4%)	15.92	2.42–180.8	0.004^*a*^
≥18 years	36 (78%)	13 (59%)	23 (96%)	0.06	0.006–0.41	0.004^*a*^
Gender						
Female	27 (59%)	13 (59%)	14 (58%)	1.03	0.34–3.08	>0.999
Male	19 (41%)	9 (41%)	10 (42%)	0.97	0.32–2.9	>0.999
Clinical presentation						
Isolation conditions						
Sterile site infection	7 (15%)	4 (18%)	3 (13%)	1.56	0.37–6.76	0.694
Monomicrobial	24 (52%)	10 (45%)	14 (58%)	0.59	0.19–2.02	0.555
Healthcare-associated risk factors						
Intensive care unit	15 (33%)	6 (27%)	9 (33%)	0.63	0.19–2.05	0.539
Days in healthcare settings (>7 days)	28 (61%)	15 (68%)	13 (54%)	1.81	0.5–6.18	0.378
Invasive mechanical ventilation	16 (35%)	8 (36%)	8 (33%)	1.14	0.35–3.72	>0.999
Gastrointestinal surgery	4 (9%)	2 (9%)	2 (8%)	1.1	0.16–7.51	>0.999
Neutropenia (< 0.5 G/L)	3 (7%)	0 (0%)	3 (13%)	0	0–1.21	0.235
Central venous catheter	25 (54%)	12 (55%)	13 (54%)	1.02	0.33–3.03	>0.999
Parenteral nutrition	26 (57%)	13 (59%)	13 (54%)	1.22	0.38–3.64	0.774
Underlying comorbidities						
Diabetes mellitus	4 (9%)	2 (9%)	2 (8%)	1.1	0.16–7.51	>0.999
Renal failure	15 (33%)	10 (45%)	5 (21%)	3.17	0.93–11	0.116
Hematological malignancy	3 (7%)	0 (0%)	3 (13%)	0	0–1.21	0.235
Solid malignancy	5 (11%)	1 (5%)	4 (17%)	0.24	0.02–1.73	0.349
Treatment						
Fluconazole therapy	7 (15%)	3 (14%)	4 (17%)	0.79	0.18–3.28	>0.999
Probiotic administration	16 (35%)	13 (59%)	3 (13%)	10.11	2.46–37.48	0.002^*a*^
*S. “boulardii”* administration	13 (28%)	11 (50%)	2 (8%)	11	2.15–53.5	0.003^*a*^
Mortality						
30-day mortality	9 (20%)	5 (23%)	4 (17%)	1.47	0.34–5.37	0.718
Laboratory results						
Blood parameters (mean with range)						
White blood cell count (giga/L)	10.8 (0.2–39.6)	13.3 (4.8–39.6)	8.2 (0.2–19.1)	1.24	1.03–1.49	0.021^*a*^
Neutrophil granulocyte count (giga/L)	8.0 (1.9–36.2)	9.5 (1.9–36.2)	6.3 (2.4–14.6)	1.19	0.97–1.46	0.1
Lymphocyte count (giga/L)	1.9 (0.6–3.5)	2.2 (0.7–2.9)	1.5 (0.6–3.5)	1.79	0.88–3.64	0.111
C-reactive protein (mg/L)	73.2 (1–265.4)	94.1 (12.4–265.4)	53.4 (1–213.1)	1.01	0.99–1.02	0.099
Procalcitonin (ng/mL)	7.1 (0.1–89.5)	12.1 (0.2–89.5)	2.1 (0.1–9.2)	1.12	0.86–1.47	0.395

^*a*^ Significant differences.

### Clinical isolates related to baker’s yeasts

The second most frequently identified phylogenomic group among the isolates was the Mixed Origin, which was recently grouped into two clades, Mixed Origins 1 and 2 (16). In our recent phylogenomic analysis focusing on baker’s yeasts, several distinct subclades primarily associated with commercial baker’s yeast strains were delineated by Rácz et al. (23). We refer to these clades as baker’s yeasts below. Notably, of the 46 *S. cerevisiae* isolates analyzed, 15 (33%) clustered within these baker’s yeast clades, as shown in Table 2 and Table S1. Seven genomes clustered with the predominantly triploid subclade (*subclade f* in Rácz et al. [23]) of Mixed Origins 1 that contains several described clinical isolates. One genome (DE42136) was shown to be a member of a tetraploid commercial baker’s yeast group of the same clade (*subclade e* in Rácz et al. [23]) with almost exclusively commercial baker’s yeasts. Among the Mixed Origins 2 yeasts, a tetraploid group (*subclade b* in Rácz et al. [23]) contained four clinical isolates of this study, and one sample (DE27916) was related to a small subclade of baker’s yeasts named *subclade a*. Two isolates clustered with the primarily tetraploid *subclade c* of Rácz et al. (23), which is dominated by clinical samples along with baker’s and sourdough yeasts (Fig. 1A; Fig. S2). Table S1 lists the individual isolates and their phylogenomic placement. The median amount of heterozygous variant positions in the Mixed Origins 1–2 clades’ clinical isolates in this study was found to be 0.2044% (s.d. = 0.1336) (Fig. 1B), with almost homozygous isolates (>0.1%), namely DE49913 and DE37603 in *subclade f*, and DE27916 in *subclade a* (Table S1), while all other isolates had heterozygosity levels of 0.1013%–0.5327%.

Patients with such baker’s yeast isolates, belonging to any of the above-mentioned Mixed Origins clades and subclades (Table 3), were less likely to have received parenteral nutrition (33% vs 68%, OR 0.24, 95% CI 0.07–0.83, *P* = 0.055) or to have had central venous catheters in place (33% vs 65%, OR 0.28, 95% CI 0.08–0.94, *P* = 0.063), with both trends nearing statistical significance. A significantly lower proportion of patients in the baker group had renal failure (7% vs 45%, OR 0.09, 95% CI 0.01–0.65, *P* = 0.0171). Probiotic use (13% vs 45%, OR 0.19, 95% CI 0.04–0.9, *P* = 0.0491) and *S. cerevisiae* var. “*boulardii”* administration (7% vs 39%, OR 0.11, 95% CI 0.01–0.87, *P* = 0.0351) were both significantly less frequent among patients with baker’s yeast isolates. Moreover, there was no significant difference in 30-day mortality between the groups (7% vs 26%, *P* = 0.235). Regarding laboratory parameters, patients with baker’s yeast isolates showed significantly lower C-reactive protein levels compared to those with non-baker isolates (mean 19.6 mg/L vs 98.0 mg/L; OR 0.97, 95% CI 0.95–0.99, *P* = 0.012). Baker’s yeast showed overall similar antimycotic susceptibility patterns as the whole 46 samples in this study, while showing higher MIC_50_ values for amphotericin B (16 mg/L compared to 8 mg/L) and micafungin (0.5 mg/L compared to 0.125 mg/L) as detailed in Table 1. Among the baker’s yeast-related isolates, only DE7856, an isolate from an abdominal wound, showed invasivity (Table S1).

**TABLE 3:** Patient statistics comparing isolates of the baker’s yeast clades with other phylogenomic groups

Variables	Total	Baker’s yeast	Non-baker’s yeast	Odds ratio	95% confidence intervals	*P*-value
	46 (100%)	15 (33%)	31 (67%)			
Demographic						
Age						
<18 years	10 (22%)	1 (7%)	9 (29%)	0.17	0.01–1.14	0.132
≥18 years	36 (78%)	14 (93%)	22 (71%)	5.73	0.88–66.79	0.132
Gender						
Female	27 (59%)	11 (73%)	16 (52%)	2.58	0.73–8.48	0.21
Male	19 (41%)	4 (27%)	15 (48%)	0.38	0.12–1.38	0.21
Clinical presentation						
Isolation conditions						
Sterile site infection	7 (15%)	1 (7%)	6 (19%)	0.29	0.02–2.35	0.399
Monomicrobial	24 (52%)	9 (60%)	15 (48%)	1.6	0.49–5.17	0.539
Healthcare-associated risk factors						
Intensive care unit	15 (33%)	5 (33%)	10 (32%)	1.05	0.29–3.82	>0.999
Days in healthcare settings (>7 days)	28 (61%)	6 (40%)	22 (71%)	0.27	0.09–0.95	0.058
Invasive mechanical ventilation	16 (35%)	4 (27%)	12 (39%)	0.57	0.17–2.1	0.520
Gastrointestinal surgery	4 (9%)	1 (7%)	3 (10%)	0.66	0.04–4.87	>0.999
Neutropenia (< 0.5 G/L)	3 (7%)	0 (0%)	3 (10%)	0	0–2.36	0.541
Central venous catheter	25 (54%)	5 (33%)	20 (65%)	0.28	0.08–0.94	0.063
Parenteral nutrition	26 (57%)	5 (33%)	21 (68%)	0.24	0.07–0.83	0.055
Underlying comorbidities						
Diabetes mellitus	4 (9%)	1 (7%)	3 (10%)	0.66	0.05–4.87	>0.999
Renal failure	15 (33%)	1 (7%)	14 (45%)	0.09	0.01–0.65	0.017^*a*^
Hematological malignancy	3 (7%)	0 (0%)	3 (10%)	0	0–2.36	0.541
Solid malignancy	5 (11%)	2 (13%)	3 (10%)	1.44	0.23–7.67	>0.999
Treatment						
Fluconazole therapy	7 (15%)	1 (7%)	6 (19%)	0.3	0.02–2.34	0.399
Probiotic administration	16 (35%)	2 (13%)	14 (45%)	0.19	0.04–0.9	0.049^*a*^
*S. “boulardii”* administration	13 (28%)	1 (7%)	12 (39%)	0.11	0.01–0.87	0.035^*a*^
Mortality						
30-day mortality	9 (20%)	1 (7%)	8 (26%)	0.21	0.02–1.39	0.235
Laboratory results						
Blood parameters (mean with range)						
White blood cell count (giga/L)	10.8 (0.2–39.6)	8.4 (3.3–11.8)	13.0 (0.2–39.6)	0.87	0.47–1.0	0.1
Neutrophil granulocyte count (giga/L)	8.0 (1.9–36.2)	6.1 (2.4–9.2)	10.1 (1.9–36.2)	0.85	0.68–1.06	0.14
Lymphocyte count (giga/L)	1.9 (0.6–3.5)	1.6 (0.6–3.3)	1.9 (0.7–3.5)	0.71	0.32–1.57	0.392
C-reactive protein (mg/L)	73.2 (1.0–265.4)	19.6 (1.0–94.9)	98.0 (1.0–265.4)	0.97	0.95–0.99	0.012^*a*^
Procalcitonin (ng/mL)	7.1 (0.1–89.5)	1.0 (0.1–5.1)	9.8 (0.1–89.5)	0.72	0.42–1.22	0.22

^*a*^ Significant differences.

### Other clades among clinical samples

Additional clades represented among the clinical isolates in this study were sporadic and therefore not subjected to detailed statistical analysis (Fig. 1A; Fig. S2). The diastatic ale yeast clade (formerly Beer 2 or Mosaic Beer, more recently termed the Belgium Beer 1 clade) was identified as the origin of a single clinical isolate (DE31630).

The Commercial Wine clade was represented in our data set by a single isolate. This lineage includes several well-known Champagne yeast strains, and the clinical isolate DE43763 clustered extremely closely with these, together with winery isolates from Italy and Australia. Thus, DE43763 belongs to a group of nearly identical strains that are widely used in the secondary fermentation of wines and other beverages and are available commercially (e.g., EC1118 and CBC-1). With respect to killer activity and dsRNA virus infection, DE43763 was the only isolate identified as a killer yeast, producing a pronounced inhibition zone against the sensitive control strain (Table S1). Samples DE42651 and DE52088 clustered with genomes of the AU Wine 1 clade, which predominantly comprises European, Australian, and North American wine yeasts. Isolate DE29607 was closely related to strains BME and NCYC3038, all clinical isolates in the French Dairy clade (Table S1), and showed moderate invasiveness in 3% agar YPD medium. Four additional isolates could not be assigned to any of the major *S. cerevisiae* clades; these instead clustered with genomes previously classified within the Mosaic M3 group described by Peter et al. (17). Samples DE32039 and DE11595 clustered with isolates from rotten banana in Costa Rica and plants in Russia, along with clinical and other isolates of unknown geographic origin, but showed no clear relationship to the established major clades. The other two unplaced samples, DE48316 and DE46258, were also positioned close to DE32039 and DE11595 and were nearly identical in their phylogenomic positions with respect to each other. They also displayed the same distinct banding pattern in fingerprinting analysis. Yet, they differed in heterozygosity by an order of magnitude: 0.0142% and 0.1206% heterozygosity in the case of DE48316 and DE46258 was determined, respectively (Table S1). Both were collected from throat swabs of a 21-year-old male patient with acute myeloid leukemia and acute myeloid lymphoma, sampled 3 weeks apart in 2019 (Table S1).

## DISCUSSION

*Saccharomyces cerevisiae* has traditionally been regarded as a non-pathogenic yeast, widely employed in the food industry and scientific research. However, an increasing number of clinical reports have confirmed its opportunistic pathogenic potential, particularly in immunocompromised individuals, patients admitted to intensive care units, and those receiving invasive medical interventions. In this study, we examined isolates from a single hospital obtained from a considerably broad patient population and a wide range of clinical specimen types and took the phylogenomic placement of all yeast isolates into account. Earlier studies either focused on the phylogenomics of a selected number of clinical isolates (41, 42), often from decades-old culture collections from a wide range of locations (19, 21), or presented retrospective clinical data without molecular typing and genomics (4–6, 11, 12, 43).

In this study, 46 clinical *S. cerevisiae* isolates were examined, of which *S. cerevisiae* var. “*boulardii”* (the probiotic yeast) accounted for a substantial proportion (22/46; 48%), as demonstrated by phylogenomics and fingerprinting data. Most of these probiotic-origin isolates were derived from the respiratory tract (17/22; 77%). Our data suggest that *Saccharomyces* infections and colonization—particularly those of confirmed probiotic origin—significantly affect pediatric patients (OR 15.92, *P* = 0.004; Table 2). We also report a strong correlation between probiotic exposure and subsequent *Saccharomyces* isolation (OR 10.11, *P* = 0.002 for probiotics in general; OR 11.0, *P* = 0.003 for *S.* var. “*boulardii”*; Table 2). Furthermore, two of the probiotic isolates (9%) were from fungemia cases and two more from other sterile sites in this study (Table S1). These observations align with previously reported concerns about the potential risks of probiotic use in vulnerable populations (4, 6), including children as recently reported in various case studies (e.g., references 44–46). The high prevalence of the probiotic subtype is consistent with numerous case reports of probiotic yeast-associated infections. However, the majority of earlier reports were limited to individual case studies and did not include comprehensive molecular typing or definitive identification of the probiotic subtype, leaving uncertainty as to whether the infections described were truly attributable to the administered probiotic strains. A study from a Belgian hospital by Poncelet et al. (5) recently investigated 10 cases of *S. cerevisiae* fungemia and demonstrated that most were associated with the administration of probiotics containing *S. cerevisiae* var. “*boulardii*.” The study highlighted elderly and critically ill patients when discussing probiotic-associated fungal infections and did not cover cases affecting children. Rannikko et al.’s study (47) of patients in five Finnish university hospitals (thus, covering a much larger patient population than our study) over 10 years found 46 patients with *Saccharomyces* fungemia, of which 20 (43%) used the probiotic. *Saccharomyces* subtyping was not applied. The study also reported probiotic use among 19% of patients (odds ratio 10) with non-blood *Saccharomyces* samples, comparable to our results in a single clinic. A comprehensive retrospective analysis on fungemia cases from a Missouri, USA, hospital by Wombwell et al. (4) identified fungemia in 18 probiotic recipient patients in merely 3 years, compared to the two confirmed probiotic fungemia cases reported in our study over 8 years or the 20 suspected probiotic fungemia cases in the five Finnish hospitals over 10 years by Rannikko et al. (47). The case number in the Missouri hospital amounted to 0.11% of patients receiving probiotic yeast treatment and highlighted an increase in the risk of fungemia linked to intensive care unit admission. Furthermore, a systematic review of 108 *Saccharomyces* fungemia cases worldwide also highlighted treatment with *S.* var. “*boulardii”* probiotics (*n* = 73, 68%) as a risk factor leading to fungemia (6). Based on the comparisons made here, regional differences in probiotic-related cases and the underlying factors of these may be put into focus by future studies.

Although *Saccharomyces* is widely regarded as a non-pathogenic or low-virulence organism, our data reinforce growing evidence that probiotic-related invasive infections may constitute a causally linked iatrogenic complication. While the 30-day mortality rate did not differ significantly between probiotic and non-probiotic cases, elevated inflammatory markers—such as higher white blood cell counts in the probiotic group (*P* = 0.021)—suggest a more pronounced systemic response. These findings highlight the need for stricter clinical caution when prescribing *Saccharomyces*-based probiotics, as suggested by Poncelet et al., who underline the contraindication of probiotics for elderly patients (5), Vinayagamoorthy et al. (6), who called attention to the increased risk for critically ill patients during probiotic treatment, and Li et al. (1), who in general, called attention to the often overlooked role *Saccharomyces* may play in infections of patients with advanced HIV. Reports confirming the identity of clinical isolates as belonging to the probiotic subtype, such as ours, provide valuable insights into the prevalence of probiotic yeasts in the inpatient population—particularly among children—and enable a more accurate estimation of risk factors. Importantly, such data help to contextualize case reports that focus solely on severe infection cases and often lack a broader overview of *S. cerevisiae* diversity and incidence in hospitalized patients. As our results demonstrate (Fig. S1), diagnosing a probiotic infection does not necessarily require complex bioinformatic analyses and phylogenomics but can be achieved rapidly using multiplex fingerprinting (13). It is also notable that the various var. “*boulardii”* strains marketed worldwide showed extreme similarity in our large-scale phylogeny; thus, the origin of clinical isolates cannot be tracked to a single brand or product. At the same time, several strains used in products are not yet available as short-read sequenced genomes for comparison (48). Despite the very close clustering of var. “*boulardii”* isolates, heterozygosity values varied substantially, and several clinical isolates from Debrecen showed relatively high heterozygosities, the median value for these isolates being 0.1902%. Notably, in Loegler et al.’s analysis (16), the probiotic subclade was characterized by very low heterozygosity, with a median value of 0.0146%. Further studies could include more product isolates to test for possibly more heterozygous strains of this subtype, which could help link clinical isolates with commercial strains within the var. “*boulardii*.” It can also not be excluded that the higher heterozygosity is through novel mutations in several clinical isolates.

The Mixed Origins clades, the groups of the *S. cerevisiae* species mostly associated with well known and widely used baker’s yeast strains (23), were the second most common among our samples (15/46; 33%). Their heterozygosity was somewhat lower than previously determined for the mostly polyploid Mixed Origins 1–2 clades: in Loegler et al.’s (16) analysis, the median heterozygosity value was 0.4426%, and for the Debrecen clinical isolates, this value was 0.2044%. These yeasts of the Mixed Origins clades have already been associated with infections and colonization of unknown frequency (21–23, 41, 42), and our current study highlighted that they are surprisingly diverse even among the isolates from a single hospital. Both clades of the Mixed Origins yeasts and five well-defined subgroups were identified in Debrecen. These all contain numerous, extremely similar commercial strains available to the broad public (23); thus, the origin of the clinical isolates cannot be traced to individual strains. Most of the baker’s yeast was recovered from the female genital tract (10/15, 67%), and only one was collected from a child. Isolates were mostly recovered from patients not receiving parenteral nutrition or having catheters. These observations raise the possibility that baker’s yeasts and probiotic yeasts have differing preferences in hosts and anatomical sites. Whether this may be caused solely by the differences in patients’ exposure to products containing these or by different stress tolerance capabilities and virulence factors would need further investigation.

Several other and distantly related clades were identified as the origin of clinical isolates, and altogether nine samples (20%) belonged to these sporadically present groups or were unplaced. The diastatic ale yeasts (formerly Beer 2 or Mosaic beer, recently Belgium Beer 1 clade) have almost exclusively been collected from the brewery environment, with merely four recorded isolates from a clinical setting (from the UK and from unknown locations) (16). These yeasts are known to be able to survive and multiply in packaged beer; thus, a food origin for clinical isolates may be plausible (49, 50). Surprisingly, a single isolate clustered with the Commercial Wine clade, which so far has exclusively been recovered from the winery environment and from bottled wines and sparkling wines (16–18, 50), and is widely available as active dry yeast for primary fermentation and bottle conditioning (mostly known as Champagne or Prise de Mousse yeasts). To our knowledge, tolerance of high temperatures in this clade has not been evaluated due to their use, almost exclusively at cold fermentation temperatures, but our sample showed good growth at 37°C and even retained its killer toxin-producing ability that characterizes many commercially available, very closely related strains.

A single sample in our phylogeny clustered with a clade of wine yeasts described earlier as Wine/Wild, which recently was grouped into the AU Wine 1 clade (16). This is a relatively large group of yeasts almost exclusively from the winery environment and from natural sources, with merely nine known clinical isolates from the United States and Europe (16, 17). One isolate was placed into the French Dairy clade, most well-known from fermented dairy products from Europe and Asia and includes four known clinical isolates from Europe and an undetermined location (16). The yeast samples not placed into any of the described clades also provide notable observations. Two samples from a single patient showed extremely close phylogenomic similarity and represent two isolates of the same strain, differing in heterozygosity (Table S1). To our knowledge, these yeasts present the first direct genomic evidence of a *Saccharomyces* strain’s long-term colonization in a patient. Notably, this pair of yeasts only showed distinct relatedness to previously sequenced samples, and their closest relatives were geographically distinct environmental isolates. The remaining two unplaced samples clustered with the ones from the same patient, and one of them represented a fungemia isolate from a blood culture (Fig. 1A; Fig. S2).

Regardless of phylogenomic relationships, our isolates generally lacked invasivity or complex colony morphologies that are often regarded as potential virulence traits (41, 51). Among the unplaced, the probiotic, and the baker’s yeast clinical isolates, only one sample in each group was invasive, and none showed complex colonies. Invasivity in YPD medium has not been found in our recent comparison of probiotic commercial strains and in-host-evolved clinical samples (8); thus, the current observation for the isolate DE33294 shows an interesting, potentially newly evolved trait. Baker’s yeasts lack systematic virulence factor assessment to allow for detailed comparisons with our current results.

While potential virulence factors and their differences among the major clades of clinical isolates are still not well understood in the case of this species (8, 22, 40), we may view antifungal susceptibility patterns as one of the most important phenotypic characteristics of clinical *Saccharomyces* isolates, as these directly relate to treatment options in healthcare settings. Susceptibility profiles to echinocandins in our study were consistent with published literature. Most isolates exhibited susceptibility to anidulafungin (MIC_50_: 0.5 mg/L), micafungin (MIC_50_: 1 mg/L), and caspofungin (MIC_50_: 2 mg/L). These findings are in agreement with a recent study by Górzyńska et al. (11), which reported 100% susceptibility to all echinocandins among 55 clinical isolates, based on EUCAST breakpoints. The study of Yan et al. (12) from Southwest China characterized 75 clinical isolates (only from women and children) and found markedly lower MIC_50_ values (0.12 mg/L for micafungin and caspofungin; anidulafungin was not tested).

All isolates in our study fell into the wild-type category for fluconazole (MIC ≤ 16 mg/L). However, it is noteworthy that the MIC_90_ value reached 8 mg/L, which could be considered an early warning sign. Górzyńska et al. (11) observed a potential fluconazole resistance rate of 24% among their 55 isolates, with cross-resistance noted in several cases to other azoles such as itraconazole, voriconazole, and posaconazole, while Yan et al. (12) reported an even higher MIC_90_ value of 16 mg/L for fluconazole. These findings suggest that while fluconazole remains a viable treatment option for *S. cerevisiae* infections, MIC-guided, strain-specific therapy is highly recommended to avoid therapeutic failure due to inter-strain variability. An apparent geographic variability that might be linked to the geographic distribution of clades of the species may also be taken into account.

Amphotericin B retained its efficacy against most isolates, with an MIC_50_ value of 1 mg/L; however, 15 isolates fell into the non-wild-type category (MIC > 1 mg/L). Górzyńska et al. (11) and Yan et al. (12) both described a similar susceptibility pattern (MIC_90_: 0.5 mg/L), and the former identified only three non-wild-type isolates. Nevertheless, given that *S. cerevisiae* infections frequently affect elderly and multimorbid patients, the potential toxicity of amphotericin B warrants careful consideration in clinical decision-making.

Our results highlight the importance of integrating patient data, antifungal susceptibility testing, and phylogenomics or strain typing to gain a broader understanding of how *S. cerevisiae* colonizes and infects patients in healthcare settings. Additional data enabling comparisons across geographic regions and with commercially available strains are also needed. A deeper understanding of clinical *S. cerevisiae* strains may ultimately support improved treatment strategies and promote the safer application of probiotic and other *Saccharomyces* yeasts.

## Data Availability

Raw genome sequencing files of the yeast samples are uploaded to NCBI GenBank (BioProject PRJNA1313639).

## SUPPLEMENTAL MATERIAL

Figure S1 spectrum.02750-25-s0001.tif Distinct banding pattern characteristic of probiotic isolates. Representative electrophoretic profile showing the characteristic banding pattern of probiotic-origin isolates.

Figure S2 spectrum.02750-25-s0002.pdf Searchable text of the tree with all genome names.

Table S1 spectrum.02750-25-s0003.xlsx Summary of clinical characteristics associated with *Saccharomyces* isolates, including patient-related data and isolate-specific features.

ASM does not own the copyrights to Supplemental Material that may be linked to, or accessed through, an article. The authors have granted ASM a non-exclusive, world-wide license to publish the Supplemental Material files. Please contact the corresponding author directly for reuse.
