# Supplementary figures and images for: Phylogenomic diversity of clinical Saccharomyces cerevisiae and the prevalence of probiotic-derived isolates in a tertiary care center in Hungary
Source: Microbiol Spectr. 2025 Nov 26;14(1):e02750-25. doi: 10.1128/spectrum.02750-25 (PMC12772296; doi:10.1128/spectrum.02750-25)

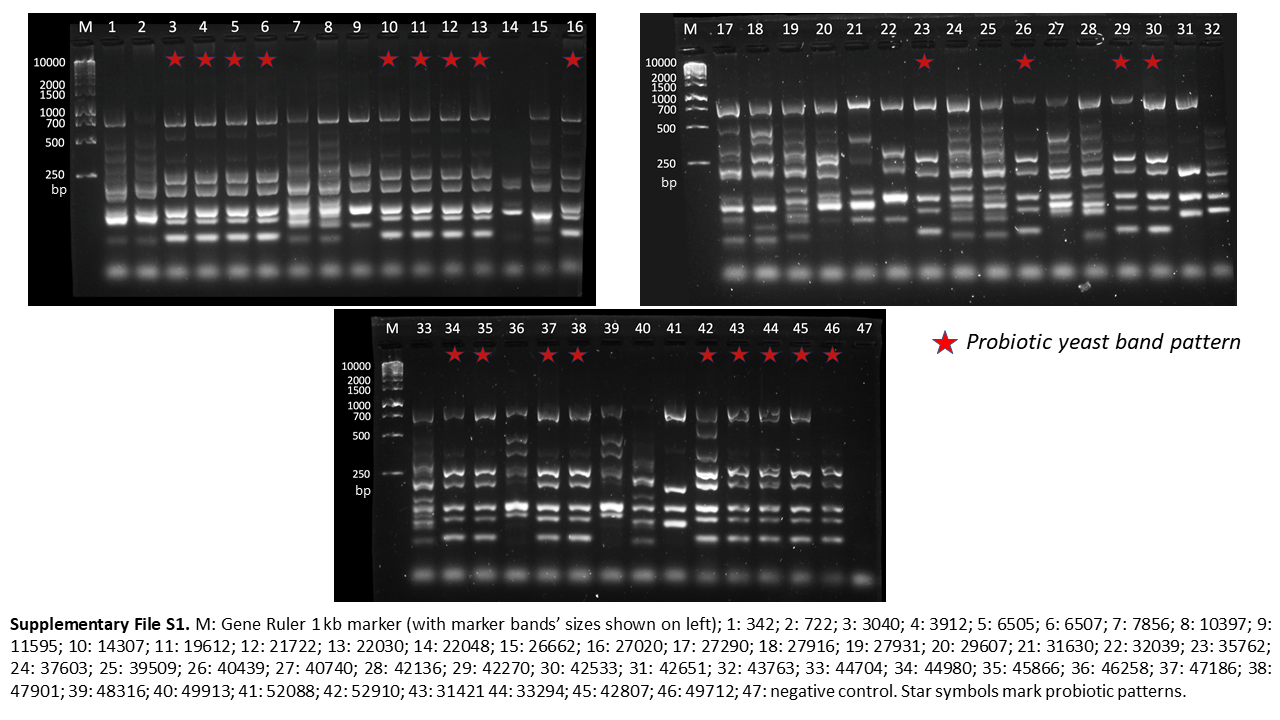

Supplement: Figure S1 — Distinct banding pattern characteristic of probiotic isolates. Representative electrophoretic profile showing the characteristic banding pattern of probiotic-origin isolates. [file spectrum.02750-25-s0001.tif]
