# Supplementary material for: Phylogenomic diversity of clinical Saccharomyces cerevisiae and the prevalence of probiotic-derived isolates in a tertiary care center in Hungary
Source: Microbiol Spectr. 2025 Nov 26;14(1):e02750-25. doi: 10.1128/spectrum.02750-25 (PMC12772296; doi:10.1128/spectrum.02750-25)

Supplementary File S2. Phylogeny of S. cerevisiae genomes. Clades are marked with various colors, clinical isolates from Debrecen are marked with red star symbols. Full green triangles mark female, empty triangles male patients. Magenta triangles mark collection from normally sterile anatomical sites.

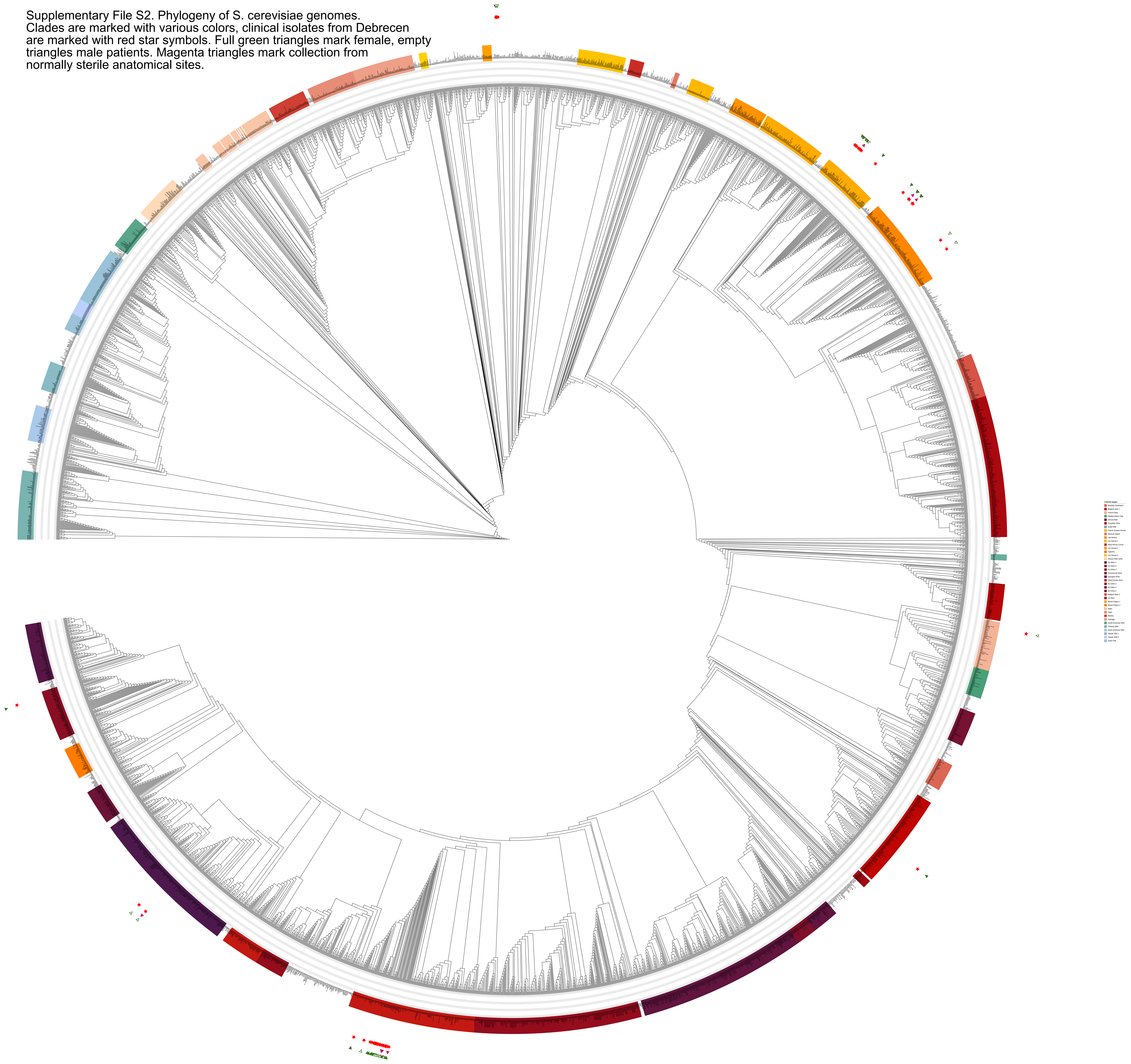

Supplement: Figure S2 — Searchable text of the tree with all genome names. [file spectrum.02750-25-s0002.pdf]
